# *BRCA1* Protein Expression in Epithelial Ovarian Cancer and Associated Clinicopathological Factors in Uganda

**DOI:** 10.1155/2024/9527113

**Published:** 2024-10-29

**Authors:** Tonny Okecha, Derrick B. Abila, Dorothy L. Nabbale, Fauz Katongole, James J. Yahaya, Robert Lukande, Sam Kalungi, Hawa Nalwoga

**Affiliations:** ^1^Department of Pathology, Uganda Cancer Institute, Kampala, Uganda; ^2^Department of Pathology, Uganda Christian University, Kampala, Uganda; ^3^Department of Pathology, College of Health Sciences, Makerere University, Kampala, Uganda; ^4^Department of Pathology, Mulago National Referral Hospital, Kampala, Uganda; ^5^Department of Pathology, Busitema University, Mbale, Uganda; ^6^Department of Pathology, School of Health Sciences, Soroti University, Soroti, Uganda

**Keywords:** *BRCA1*gene, clinicopathological characteristics, epithelial ovarian cancer

## Abstract

**Background:** *BRCA1* gene dysfunction seen in epithelial ovarian carcinomas often results from germline mutations, somatic mutations, and promoter methylation. Identification of tumors with loss of *BRCA1* protein expression has shown to have therapeutic and prognostic implications. The aim of this study was to determine the expression of *BRCA1* protein in epithelial ovarian cancer (EOC) and the associated clinicopathological characteristics.

**Methods and Results:** This was a cross-sectional laboratory-based study that used paraffin-embedded tissue blocks of patients histologically diagnosed with EOC from January 2010 to August 2018. Tissue sections were stained with hematoxylin and eosin (H&E) for histological confirmation and with immunohistochemistry (IHC) using a mouse-derived monoclonal antibody MS110 for *BRCA1* protein expression. The association between *BRCA1* protein expression and independent variables was determined using Pearson's Chi-square test. A total of 104 tissue blocks from patients with EOC were included in the study with a mean age of 48.7 ± 12.8 years. Serous tumors were the most common which comprised 74.0% (77/104) of all the tumors and majority of them 75.3% (58/77) were high grade. Loss of expression of *BRCA1* protein expression was found in 33.7% (33/98) of all the cases. There was no statistically significant association between *BRCA1* expression and age of patients, tumor grade, and histological subtype.

**Conclusion:** There is a high expression of altered *BRCA1* expression in tissues of EOC. Although it has not shown association with age of patients, histology types, and tumor grade, further studies need to assess its influence of the survival of cancer patients with EOC.

## 1. Introduction

Ovarian cancer is the eighth most common cancer and the eighth leading cause of death from cancer in women worldwide [1]. In 2020, there was an estimated 313,959 cases diagnosed worldwide with an estimated 207,252 deaths attributed to ovarian cancer. Incidence rates are highest in more developed regions, with rates in these areas exceeding 7.5 per 100,000 women and lowest in sub-Saharan Africa with rates below 5 per 100,000 women [2]. In sub-Saharan Africa, there are limited studies pertaining the prevalence of ovarian cancer, and these studies are rarer in East Africa. The annual increase in incidence of ovarian cancer has been reported at 2.5% for Mauritius, 5.3% for Mali, 3.9% for Nigeria, and at 8% for South Africa [3]. Unfortunately, there is limited epidemiological data pertaining to ovarian cancer trends, incidence, and mortality in Uganda. However, data from a population-based cancer registry, i.e., Kampala Cancer Registry, show that there were 626 documented cases of ovarian cancer over the period 1991 to 2015, with an average incidence of 6.5 per 100,000 women. The highest incidence rate was recorded during the period of 2001–2005 at 8.3 per 1,00,000 women [4].

Epithelial ovarian cancer (EOC) has been reported to account for 95% of all ovarian cancers, with 11%–90% of the EOC associated with alterations in the expression of *BRCA1* protein due to mutations in the *BRCA1* gene [5]. *BRCA1* is a tumor suppressor gene located on the long arm of Chromosome 17 at Position 21 and plays a major role in preserving chromosomal stability. *BRCA1* mutations arise from a variety of mechanisms including germline, somatic mutation, and promoter hypermethylation [6]. These mutations significantly increase a woman's risk of developing breast and/or EOC. Most of the available information relates to *BRCA1*-linked disease because *BRCA1* germline mutations are approximately four times more common in ovarian cancer patients than *BRCA2* mutations [7]. *BRCA1*-related EOC represents an entity of ovarian cancers implicated in some EOC cases. These tumors have a different therapeutic and prognostic profile as opposed to the non–*BRCA1*-related cancers, since they exhibit differential response to chemotherapy and have a better 5-year survival rate. *BRCA1* mutations can be readily assessed using immunohistochemistry (IHC). *BRCA1* protein IHC is regarded as an inexpensive and rapid initial screen to detect *BRCA1* protein expression in EOC. Altered *BRCA1* protein expression as detected by IHC correlates with the presence of somatic, germline, and methylation mutations in the gene [8].

Unfortunately, there is limited data pertaining to the prevalence of *BRCA1*-related EOC as a distinct entity in East Africa and Uganda. Therefore, this study aimed to determine the expression of *BRCA1* protein in EOC and its association with clinicopathological characteristics.

## 2. Methods, Study Design, and Setting

This was a laboratory based cross-sectional study conducted at the department of pathology, Makerere University in Kampala, Uganda. The department specifically serves the roles of teaching, research, and offering diagnostic biopsy and autopsy services for the most part of the country. It receives and processes an average of 5,000 tissue specimens per year from across the entire country.

### 2.1. Study Population

We included histologically confirmed cases of EOC from January 2010 to August 2018. Only cases from the archived formalin fixed paraffin embedded (FFPE) tissue blocks of patients with sufficient tissue for IHC and hematoxylin and eosin (H&E) staining were included in the analysis. In addition, only patients who were naive for chemotherapy were included. All cases with FFPE tissue blocks that had extensive necrosis on H&E, and extensively damaged tissue blocks as well as cases with ovarian cancer but not epithelial were excluded.

### 2.2. Sample Size and Sampling Procedure

A total of 104 cases were included in the analysis. Of the 104 cases, the incisional biopsies accounted for majority 74.0% (77/104) of the cases and the remaining 26.0% (27/104) were oophorectomies. Convenience sampling method was used to obtain the cases, and cases were recruited consecutively using the selection criteria until the required sample size was obtained. Histology laboratory numbers and other information on histology request forms were used to retrieve the FFPE tissue blocks of cases meeting the inclusion criteria.

### 2.3. Tissue Processing and H&E Staining

Retrieval of the FFPE tissues blocks was done by a laboratory technician. The retrieved FFPE tissue blocks were trimmed and cut into four microns thickness, and two sets were cut from each FFPE tissue block. One set was prepared for H&E staining, while the second set was for IHC staining. The sections were stained using the routine H&E staining method by three researchers. The H&E tissue slides were examined by three pathologists (TO, HN, and RL) using Olympus BX50 microscope with a field diameter of 0.52 mm. Grading of the tumors was done based on the Silverberg grading system [9, 10].

### 2.4. *BRCA1* Protein IHC Staining

For IHC, the 4-micron thick sections upon confirmation on H&E were cut from paraffin-embedded blocks, which contained representative histology of EOC. Antigen retrieval was done using a Decloaking chamber SP1 (temperature 125° for 35 s) and SP2 (temperature 90° for 20 s). A commercially available epitope retrieval solution, pH 6 from Leica Biosystems, Germany, was used. Endogenous peroxidase activity was blocked by incubating sections with peroxidase for 5 min. The primary antibody used in the study was mouse monoclonal antibody (MS110) to *BRCA1* (Medaysis, USA), Catalog no: MC0504, Lot no: MC05040819. The dilution was optimized at 1:25. Sections were incubated with the primary antibody at room temperature for 1 h.

Sections were then washed in Tris buffer solution (TBS) wash buffer for 5 min and incubated with Novolink (Leica Biosystems) post–primary antibody for 30 min. They were then washed with TBS and Incubated with Novolink polymer for 30 min. They were stained with a detection system chromogen 3,3′-diaminobenzidine (DAB) (Leica Biosystems) for 1 min, washed with tap water, and stained with Harris hematoxylin for 1 min. The positive control used was breast cancer tissue with known immunoreactivity for *BRCA1* protein. Replacing the primary antibody with buffer solution served as the negative control.

Evaluation of the IHC-stained slides was done using quantitative visual assessment method. Immunoreactivity was characterized by brown staining of the nucleus. Cases were considered to have positive *BRCA1* protein expression when at least 10% of the tumor cells nuclei were stained which was obtained by dividing the positive tumor cells over 200 tumor cells in at least five separate locations throughout the specimen [11]. Cytoplasmic staining was also scored but not considered as positive. The intensity of staining was scored as weak, moderate, or strong. Evaluation and scoring of the IHC-stained slides was done by two independent pathologists. For cases where the two pathologists disagreed, consensus was reached by examining the cases on a multiheaded microscope by both pathologists.

### 2.5. Data Analysis

The collected data were double cross-checked and edited for any mistakes. Analysis was performed using SPSS version 22.0. The statistical association between *BRCA1* protein expression with the clinicopathological features was assessed using Pearson's Chi-square statistical test. *p* value less than 0.05 was considered statistically significant.

## 3. Results

### 3.1. Demographic and Histological Characteristics

Table 1 shows distribution of EOCs by age, histological type, and grade. A total of 104 FFPE tissue blocks from patients with EOCs were analyzed for histological type, tumor grade, and *BRCA1* protein expression in this study. The mean age of the patients was 48.7 ± 12.8 years (range: 19–75 years). Slightly over half, 52.9% (55/104) of the patients were 50 years of age and above. Histologically, serous tumors were the vast majority 74.0% (77/104) of the cases. Concerning tumor grading, most of the nonserous tumors 44.5% (12/27) and majority of serous tumors 75.3% (58/77) had high grade, respectively.

### 3.2. IHC Staining for *BRCA1* Protein

**Figure 1:**
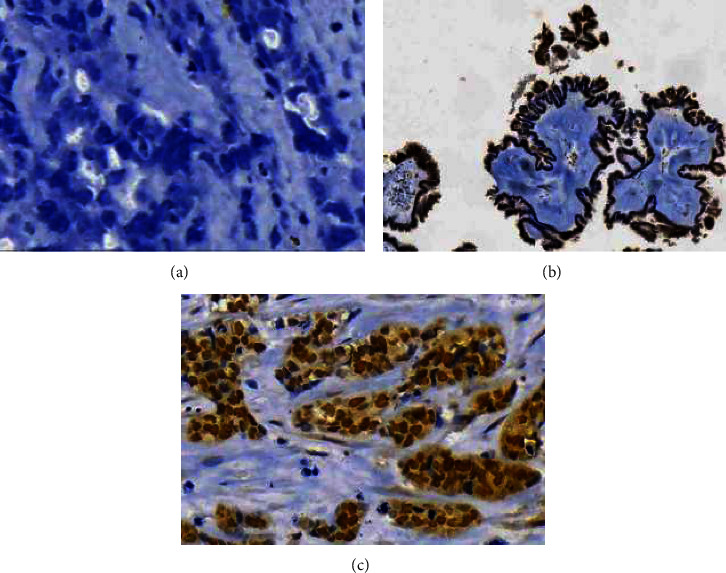
(a) Photomicrograph showing negative staining for BRCA1 protein, (b) photomicrograph showing normal 10% nuclear staining for BRCA1 protein, and (c) photomicrograph showing > 10% strong and diffuse nuclear staining of BRCA1 protein (immunohistochemistry staining, × 200).

**Figure 2:**
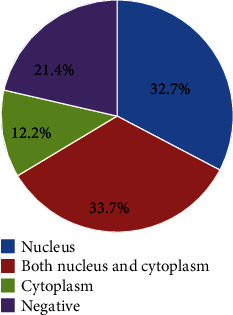
BRCA1 protein in staining different sites of the tumor cells association of clinicopathological features with BRCA1 protein expression.

A total of 98 cases were included for IHC staining of *BRCA1* IHC staining due to insufficient *BRCA1* antibody to stain all the 104 cases. The prevalence of loss of *BRCA1* protein expression among EOC cases in this study was 33.7% (33/98). Nuclear staining was 32.7% (32/98) of the cases, and 33.7% (33/98) had both nuclear and cytoplasmic staining. Among the cases with retained *BRCA1* expression, 21.5% (14/65) had strong nuclear staining (Figures 1(b) and 1(c)), 46.2% (30/65) showed moderate staining intensity, while 32.3% (21/65) had mild staining intensity (Figure 2).

The association between *BRCA1* protein expression with independent variables is presented in Table 2. There was no statistically significant difference in expression of the *BRCA1* protein with the age of the patients (95% CI = 0.42–2.24, *p*=0.944), histological types (95% CI = 0.70–1.52, *p*=0.867), and tumor grade for nonserous EOC cases (95% CI = 0.51–4.41, *p*=0.482) as well as serous tumors (95% CI = 0.51–4.41, *p*=0.482). Also, age of archival of the FFPE tissue blocks was not associated with *BRCA1* protein expression despite showing a trend of loss of expression with increase of the archival age of the tissue blocks (95% CI = 0.25–1.49, *p*=0.278).

## 4. Discussion

This study was undertaken to determine *BRCA1* protein expression in EOC and the associated clinicopathological factors. The mean age in this study agrees with previous studies conducted on patients in other African countries [12, 13]. However, several previous studies in the Caucasian populations in Europe and North America report a mean age at diagnosis of 58 years, which is a decade higher than African patients [14, 15]. The peak age of occurrence of EOC was between fifth and sixth decade. This finding also agrees with various studies carried out on patients with EOC in other African countries [13, 16].

The finding of serous carcinomas as the most common histological subtype is consistent with several previous studies that found it as the most common in Africa and other countries [13, 17, 18]. However, a systematic review study regarding global distribution of the histological types of EOC showed that the relative frequencies of subtypes varied across countries which may reflect geographical and ethnic diversity [19]. As such, the current study found that the endometrioid type was the second most common type of tumor, which is also in agreement with the previous studies [17, 20]. However, other previous studies showed that mucinous tumors are the second most common in Africa [13] and Europe [21]. The pattern of distribution of histological types in the current study and the previous studies supports the conclusion that EOC is a heterogeneous disease with a heterogeneous distribution pattern.

Most tumors in the present study were of Grade II followed by Grade I, and the findings were consistent with a few previous studies carried out elsewhere [22, 23]. However, this finding mostly result differs from the previous studies in Africa and other continents where Grade 3 is the commonest grade reported at histology [11, 13, 24]. These differing findings could be explained by the difference in the tumor biology across different geographical areas since the grading systems employed in all the studies were similar.

In the current study, about 34% of the EOCs had altered protein expression (complete loss or reduced expression) which is within the range (12.1%–90%) of *BRCA1* protein expression in EOC reported previously [24–27]. The reasons for this wide range of altered *BRCA1* protein expression could partly be explained by differences in methodology such as sample size, IHC assay conditions, interpretation of staining, and the stage of disease. The varying tumor biology coupled with stage of disease and treatment could also contribute to this difference. A variation in the proportion of *BRCA1* gene mutations in the Ugandan patients versus other populations could also be postulated as a plausible explanation. However, further studies are needed to validate this possibility. The prevalence of altered *BRCA1* protein expression in this study is also high in comparison to the proportion of patients that show only germline *BRCA1*-mutated EOCs, which is estimated to be 5%–15% [28, 29]. This is because *BRCA1* IHC detects tumors with loss of function of *BRCA1* gene due to germline mutations, somatic mutations, and epigenetic changes of the *BRCA1* gene region. Therefore, this higher percentage alludes to the fact that IHC detects alteration in the *BRCA1* expression regardless of the cause and not limited to germline mutations. In the present study, a proportion of the tumors (12.2%) showed cytoplasmic expression which is similar to previous studies carried out in both normal ovarian tissue and ovarian cancer.

A study indicated that cytoplasmic expression detected in normal ovarian tissues represents the splice variant protein losing most of exon 11 recognized by monoclonal antibodies against *BRCA1* gene [30]. This is supported by an earlier study done which showed that among the different splice variants of *BRCA1* gene, the *BRCA1*-delta 11b, which lacks most of Exon 11, was localized in the cytoplasm instead of the nucleus. In addition, a study by Rodriguez et al. showed that mutations that disrupt or delete the C-terminal BRCT domains, but not other regions of *BRCA1* gene, caused significant relocalization of *BRCA1* gene from nucleus to cytoplasm [31]. Wilson et al. therefore suggested that this splice variant of *BRCA1* protein, lacking most of Exon 11 may have distinct roles in cell growth regulation and tumorigenesis in ovarian cancer [32]. This is similar to what Rakha et al. suggested that *BRCA1* gene cytoplasmic expression may play an important role in breast cancer [33]. Taken together, these findings may indicate that cytoplasmic expression of *BRCA1* protein in EOC has a role in tumorigenesis. Thus, the role of *BRCA1* protein cytoplasmic staining in EOC as seen in this study requires further probing.

Concerning association of *BRCA1* protein expression with clinicopathological factors, it was found that there was no statistical association between *BRCA1* protein expression and age, which was supported by findings from another study with a similar sample size (*n* = 99), was also employed [26]. Contrary to our findings, most previous studies carried out have concluded that loss of *BRCA1* protein expression is associated with a younger age of onset of EOCs regardless of the type of mutations [11, 34, 35]. The difference in these results could be attributed to a smaller sample size employed in the study as opposed to the large-scale studies.

Tumor grading for both serous and nonserous histological subtypes of the EOCs was not associated with loss of *BRCA1* protein expression. This is in keeping with the findings in the studies of Thrall et al. [24] and Lesnock et al. [11]. However, in the study of Zheng et al., there was an association between *BRCA1* protein expression and tumor grade of the EOCs [36]. The reduced rate of *BRCA1* protein expression concurs with the fact that high-grade EOCs are associated with a poor prognosis. Although this is also against the finding in the study of Lesnock et al. in which it was observed that reduced loss of *BRCA1* protein expression was favoring a better prognosis among patients with EOCs [11]. This converse association of *BRCA1* expression with high-grade EOCs indicates that there is a possibility that pathogenesis of EOCs depends on other molecular mechanisms other than *BRCA1* mutation alone. Furthermore, in the study of Lesnock, it was found that loss of *BRCA1* protein expression was associated with a better prognosis of patients with EOCs [11].

Histological subtypes of EOC was also not associated with *BRCA1* protein expression, which is similar to the finding in another previous study [11]. But a positive relationship between histological types of EOC and *BRCA1* protein expression was reported in the study of Thrall et al. [24]. Variation in the sample size and sampling approach could explain the discrepancy of the association between *BRCA1* protein expression and histological subtypes of the EOCs. In addition, this may also be explained by the fact that, development and progression of EOCs depend on different mechanisms apart from *BRCA1* mutation and promotor hypermethylation [37].

Some of the study limitations include difficulty in obtaining the tissue blocks from the archives due to poor storage; despite availability of request, forms could have created a selection bias. Since the study employed use of archival material, inappropriate fixation could have affected antigen retrieval during IHC. We recommend studies with a larger sample size to clarify the association of *BRCA1* with the clinic-pathological characteristics of EOC. We also recommend a prospective study to assess the association between *BRCA1* protein expression with overall survival and response to different chemotherapy modalities in ovarian cancer patients in Ugandan patients. In addition, *BRCA1* IHC could be routinely performed on all EOC patients.

## 5. Conclusion

There is a high expression of altered *BRCA1* expression in tissues of EOC. Although it has not shown association with age of patients, histology types, and tumor grade, further studies need to assess its influence of the survival of cancer patients.

## Figures and Tables

**Table 1:** Demographic characteristics of the patients with epithelial ovarian cancer (*N* = 104).

Variables	Frequency (*n*)	Percentage (%)
Age (years)		
< 20	1	1.0
20–29	8	7.7
30–39	11	10.6
40–49	29	27.9
50–59	30	28.8
60–69	18	17.3
70–79	7	6.7
Histological type		
Serous	77	74.0
Endometrioid	17	16.3
Mucinous	5	4.8
Transitional	3	2.9
Clear cell	1	1.0
Oncocytic	1	1.0
Tumor grading for nonserous EOCs		
Grade 1	10	37.0
Grade 2	5	18.5
Grade 3	12	44.5
Tumor grading for serous EOC		
Low grade	19	24.7
High grade	58	75.3

**Table 2:** Association between BRCA1 protein expression with age, histopathologic types, and tumor grade (*N* = 98).

Variables	BRCA1 protein expression		
	Normal: *n* (%)	Loss: *n* (%)	95% CI	*p* value
Age (years)			0.42–2.24	0.944
< 50	32 (66.7)	16 (33.3)		
≥ 50	33 (66.0)	17 (34.0)		
Histopathologic type			0.70–1.52	0.867
Serous	46 (64.8)	25 (35.8)		
Endometrioid	11 (64.7)	6 (35.3)		
Mucinous	5 (100.0)	0 (0.0)		
Transitional	2 (66.7.0)	1 (33.3)		
Clear cell	0 (0.0)	1 (100.0)		
Oncocytic	1 (100.0)	0 (0.0)		
Tumor grade for nonserous EOCs			0.61–2.01	0.349
Grade 1	7 (70.0)	3 (30.0)		
Grade 2	3 (60.0)	2 (40.0)		
Grade 3	5 (41.7)	7 (58.3)		
Tumor grading for serous type of EOC			0.51–4.41	0.482
Low grade	11 (57.8)	8 (42.2)		
High grade	35 (57.7)	17 (42.3)		
Age of archival tissue blocks (years)			0.25–1.49	0.278
≤ 2	27 (73.0)	10 (27.0)		
> 2	38 (62.3)	23 (37.7)		

## Data Availability

The dataset used to prepare this paper is available upon request from the corresponding author.
